# Genus Datura: An Exploration of Genetic Alterations, Bioactive Compounds, and Pharmacological Activity

**DOI:** 10.3390/plants14142244

**Published:** 2025-07-21

**Authors:** Khoirunnisa Assidqi, Nesti Fronika Sianipar, Dave Mangindaan, Chukwunwike Uchenna Enyi

**Affiliations:** 1 Biotechnology Department, Faculty of Engineering, Bina Nusantara University, Jl. KH Syahdan No. 9, Jakarta 11480, Indonesia; khoirunnisa.assidqi@binus.edu; 2 Food Biotechnology Research Center, Bina Nusantara University, Jl. KH Syahdan No. 9, Jakarta 11480, Indonesia; 3 Waste-Food-Environmental Nexus Research Interest Group, Bina Nusantara University, Jl. KH Syahdan No. 9, Jakarta 11480, Indonesia; 4 Civil Engineering Department, Faculty of Engineering, Bina Nusantara University, Jl. KH Syahdan No. 9, Jakarta 11480, Indonesia; 5 Professional Engineering Program Department, Faculty of Engineering, Bina Nusantara University, Jl. KH Syahdan No. 9, Jakarta 11480, Indonesia; 6 Department of Food Science and Technology, University of Agriculture and Environmental Science, Umuagwo, P.M.B., Owerri 1038, Imo, Nigeria; nnasedo@gmail.com

**Keywords:** pharmacological activity, bioactive compound, morphological, molecular, environmental conditions, genus *Datura*

## Abstract

The genus *Datura* L. has pharmacological activities due to its source of bioactive compounds. The effects of bioactive compounds can vary depending on species, geographical location, and environmental conditions. The purpose of this review is to summarize the most recent progress and to provide a comprehensive overview of studies concerning genetic alteration and bioactive compounds in the genus *Datura*, based on Scopus publications between 2015 and 2025. Throughout history, the genus *Datura* (Solanaceae) contains nine species of medicinal plants. A key component of elucidating the diversification process of congeneric species is identifying the factors that encourage species variation. A comparative gene family analysis provides an understanding of the evolutionary history of species by identifying common genetic/genomic mechanisms that are responsible for species responses to biotic and abiotic environments. The diverse range of bioactive compounds it contains contributes to its unique bioactivity. *Datura* contains tropane alkaloids (such as hyoscyamine and scopolamine), datumetine, withametelin, daturaolone, and atropine. Several compounds have been isolated and refined for use in treating various conditions as a result of recent progress in therapeutic development. Daturaolone, for example, is used to treat certain neurological disorders. In addition to providing renewed opportunities for the discovery of new compounds, these advancements also provide insights into the genetic basis for their biosynthesis. Our discussion also includes pitfalls as well as relevant publications regarding natural products and their pharmacological properties. The pace of discovery of bioactive compounds is set to accelerate dramatically shortly, owing to both careful perspectives and new developments.

## 1. Introduction

Genus *Datura* L. is a plant from the family Solanaceae [1]. A majority of the organs of the *Datura* plant contain compounds that can be used medically. There are several species of *Datura*, but especially from *Datura metel* L. and *Datura innoxia* Mill. are often cultivated for pharmaceutical purposes. All of these species contain tropane alkaloids, including hyoscyamine and scopolamine [2]. Based on this information, it can be inferred that *Datura* L. contains a variety of important compounds that are potentially beneficial to human health. Traditional medicine finds *Datura* species attractive due to their phytochemical composition. Alam et al. [3] reported the presence of triterpenoid, steroid, flavonoid, phenolic, tannin, saponin, and alkaloid compounds in *Datura* flower extract. *Datura* L. is also reported to contain withanolide compounds, which are found throughout the plant but are mostly concentrated in the leaves and flowers [4]. It has been demonstrated that withanolides can reduce the growth of cancer cells and reduce inflammation by blocking the NF-κB signaling pathway (a transcription factor that regulates gene expression for the innate immune response) [5,6]. *Datura* species have been extensively studied, and extracts from various parts of the plants have been shown to have anti-inflammatory, anticancer, antibacterial, antiviral, and antidiabetic properties [7,8,9,10,11]. An extract of *D. metel* L. leaves produces various sesquiterpenoids, and these compounds inhibit lipopolysaccharide-induced inflammation in RAW264.7 cells [12]. The toxic properties of *D. metel* L. extract have been shown to increase mortality in larvae of *Artemia salina* when the concentration of *Datura* extract is increased [13]. *D. stramonium* L. seed and leaf extract demonstrates antimicrobial properties against *Escherichia coli*, *Salmonella typhimurium*, and *Staphylococcus aureus* [2,14]. According to Sharan et al. [14], *D. metel* L. leaf extract is capable of reversing hypermethylation of the *CADM1* and *SOCS1* genes in cervical cancer cells, and it also has analgesic properties in Wistar rats that result in temporary loss of consciousness [15].

*Datura* L. is a genus of flowering plants with trumpet-shaped flowers, including *D. stramonium* L., *D. metel* L., *D. innoxia* Mill., and *D. ferox* L. [16,17,18]. There are several names assigned to *Datura* in different countries, such as “Kecubung” in Indonesia, “Yangjinhua” in China, and “Lingxiaohua” in Russia [19] and “Jimson weed” in the United States [20]. It is a perennial plant that can reach 1.5 m in height, flowers all year round, has aromatic foliage, likes moist soil, and has dark green leaves [1]. Recent research on the diversity of thorn apples has been published in India, where four different types of thorn apples can be identified according to flower morphology, including purple, purplish white, white, and yellow [1]. Plants of the *Datura* genus are well suited to grow in soil that is rich in nutrients [21] as well as receiving a sufficient amount of light [22]. Various developments have contributed to the discovery of genetic diversity in *Datura* species. There is a wide range of *Datura* species that are found throughout several countries, including the United States [23], India, China, the Philippines [24], and other countries, including those spread throughout several areas of Indonesia [25].

*Datura*’s genetic diversity can be attributed to several factors, including introduction, environmental changes, and gene recombination because of plant crossing [26]. Plants are susceptible to genetic improvement depending on the degree of genetic variability in the population being evaluated [27]. Understanding *Datura* genetics is an essential step in conservation and genetic improvement efforts to obtain *Datura* species that can be utilized for medical purposes. Despite this, there is still a lack of information regarding the genetic alteration that affects metabolic regulation, and the composition of bioactive compounds obtained from *Datura* plants. This review report highlights current research trends, emphasizing promising bioactive compounds with potential pharmaceutical properties, pharmacological activities, and genetic alterations across *Datura* species. The findings will help us better understand the plant’s potential for medicinal applications and sustainability. Identifying genetic variations can enable researchers to develop strategies for improving *Datura* plants’ beneficial properties.

## 2. Materials and Methods

Our literature review was accomplished using the Scopus database (http://scopus.com, accessed on 5 April 2025). Although this review was non-systematic, we evaluated whether peer-reviewed articles were chosen based on the presentation of clear methodologies, the reporting of sufficient data, and the presentation of specific results related to genetic changes and bioactive compounds related to the genus *Datura*. Plant science and pharmacology were selected based on Scopus’ extensive peer-reviewed literature coverage. According to Bass et al. [28], Scopus has a robust content curation process supervised by the Content Selection and Advisory Board. To identify bioactive compounds, we employed a combination of keywords, including “genetic alteration”, “genus *Datura*”, “secondary metabolites”, and “bioactive compounds”. To gather information on genetic variability specifically, we conducted an additional search using related terms, ensuring that relevant studies were included. The focus was on original research articles published between 2015 and 2025, a period during which there has been a growing interest in the biological activities of *Datura* species. Phytochemical investigations of *Datura* and their relationship with genetic variability were selected based on relevance to phytochemical research. A review of the collected literature was conducted to extract and analyze data regarding *Datura* species and parts used, pharmacological activities, phytochemical compounds, biological mechanisms and effects, as well as insights into genetic modification. This comprehensive approach allowed us to highlight key findings and gaps in the current research. The analysis also emphasized the potential of *Datura* species in developing new therapeutic agents and the importance of further genetic studies to enhance their bioactive properties.

## 3. Genus *Datura*: Morpho-Taxonomy

**Figure 1:**
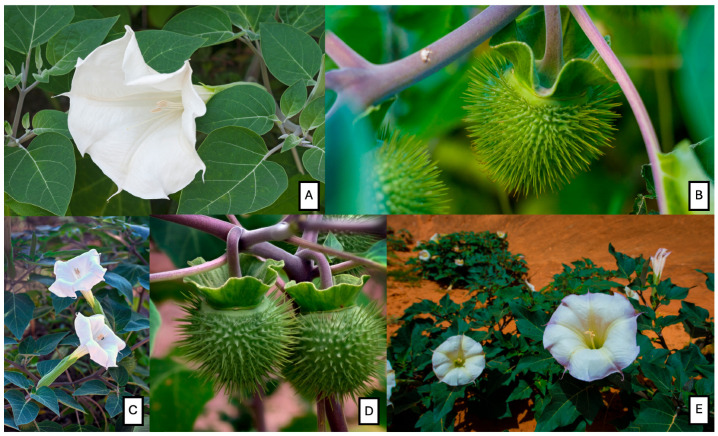
(**A**) *Datura metel* with white flower; (**B**) *Datura metel* with spiny fruit; (**C**) *Datura innoxia* L. with white flower; (**D**) *Datura innoxia* with spiny fruits; (**E**) *Datura discolor* with flower. These images were purchased and licensed through Shutterstock.com.

The *Datura* species grow as annuals or perennials, depending on the geographic region. Flowers are trumpet-shaped, leaves are often malodorous, and fruits are spiny, except for the smooth-fruited *Datura ceratocaula* [29,30]. A representative morphology of *D. metel*, *D. innoxia*, and *D. discolor* is shown in Figure 1. As a “nightshade”, *Datura* belongs to the family Solanaceae and subfamily Solanoideae, as well as to the tribe Datureae [30]. Originally known as Stramonium, Linnaeus renamed it *Datura*, a Latinization of the Sanskrit dhattura, in 1737. According to Jiménez-Lobato et al. [26], *D. stramonium* originated in North America (Mexico and the southern states) and spread to other countries by insects. Plants grown from 1 to 1.5 m high may be large, annual herbs, shrubs, or small trees. The leaves of this plant are alternate, oval, petiolate, dark green in color, and grow up to 20 cm tall. The fruit is a capsule 5 cm long and contains small, brownish, and black seeds. These fruits are often referred to as “thorn apples” due to their prickly exterior. Solitary funnel-shaped flowers, 2.5 to 12.5 cm long, with white, yellow, pink, or red petals, bloom in the late spring [31].
Taxonomic Classification: [32,33]
KingdomPlantaeSubkingdomTracheobiontaSuperdivisionSpermatophytaDivisionMagnoliophytaSubdivisionAngiospermaeClassMagnoliopsidaSubclassAsteridsOrderSolanalesFamilySolanaceaeGenus*Datura*Species*Datura* sp.

This study has identified several species in the genus *Datura*, including desert thorn apple (*D. discolor*), downy thorn apple (*D. innoxia*), *D. kymatocarpa*, *D. lanosa*, *D. metel*, *pruinose* (pruinose thorn apple), *reburra*, *ceratocaula*, *quercifolia*, sacred datura, and *wrightii* [16,34]. According to the literature, there are generally nine species of *Datura*, but there have been reports of up to fifteen species. A wide variety of naturalized species of *D. stramonium* (Jimsonweed, section Stramonium) are found throughout the world. In this species, there are four varieties (vars. stramonium, tatula, godronii, and inermis) which differ in their flower and fruit colors and morphologies [35]. *Datura ferox* (fierce thornapple) is grouped with Jimsonweed in the same section (*stramonium*). The American *Datura quercifolia* is an oak-leaved shrub that has not been extensively studied, like *Datura discolor*. This is closely related to *D. discolor*, *D. innoxia* (often spelled innoxia, Latin for “innocent” or “harmless”), which is native to the Americas. Despite the fact that *Datura wrightii* (“Wright’s *Datura*”) is often polymorphic, it is easily differentiated from *D. innoxia* [36]. The species *Datura ferox* (fierce thornapple) is found in the same section as Jimson weed (Stramonium). *D. metel* is also closely related to *D. innoxia* and *D. wrightii* (and is classified in section Dutra), and it is a cultivated ornamental variety, including alba, fastuosa, rubra, metel, and muricata [37]. The yellowish-white flowers of *Datura leichhardtii* (section Dutra) are characterized by two subspecies (*leichhardtii* and *ssp*. *pruinosa*).

Several studies of *Datura* have demonstrated that itis a hermaphrodite plant species [38]. It also produces fruits of 3 to 8 cm in diameter [39]. A *Datura* plant’s first flower buds appear 8–9 weeks after seed germination [40]. The composition of alkaloids appears to be influenced significantly by geography. This appears intuitive at first glance, as the different climates, altitudes, insect populations, and local microenvironments of different areas could impart distinct selection pressures on plants. *Datura* plants of various geographical locations were evaluated by Alexander [41] for their alkaloid contents. Alkaloid composition varies dramatically among them. For example, Berkov and colleagues [35,42] noticed that both *D. stramonium* and *D. innoxia* (roots, leaves, and seeds) had different alkaloid patterns when they were grown in Egypt (versus grown in Bulgaria). *D. kymatocarpa* and *D. reburra* are usually described as conspecific with *D. discolor*, although phenetic comparisons suggest that they are all disparate species [43]. A variety of species may also be classified as a different species (e.g., *D. fastuosa* for *D. metel* var. fastuosa, or *D. tatula* for *D. stramonium* var. tatula). Previously, members of the arboreous South American genus Brugmansia (the fourth section of the tribe Datureae) were classified under the *Datura* genus. Nevertheless, these two genera have been recognized to be morphologically distinct since the 1970s [44]. This reclassification was based on differences in flower structure, seed dispersal mechanisms, and genetic evidence. Despite their similarities, *Brugmansia* species are typically larger, have pendulous flowers, and are perennial, while *Datura* species are smaller, have erect flowers, and are mostly annual plants.

## 4. Species-Level Genetic Changes in Datura

An overview is provided in this review study of the reasons for genetic changes or increased variability in *Datura* species. It has been demonstrated in this study that genetic variability in *Datura* is primarily caused by natural factors such as natural selection from environmental influences and predators or pests in the *Datura* growing area, the process of plant crossing, and the induction of diversity through genetic transformation and tissue culture, as indicated in Table 1. Several environmental factors influence the growth of plants, including soil structure and altitude. Stressed environmental conditions cause *Datura* species to modify their growth patterns. The distribution of *Datura* species is also affected by differences in altitude. *Datura metel* species tend to be found more frequently in highlands than in lowlands, whereas *Datura stramonium* is most commonly found in lowlands and rarely in highlands [45]. Several pollinators, such as bees and moths, can contribute to genetic changes in *Datura* species. The rate of outcrossing in *Datura* was found to be 1.3% in a research study conducted by Kleunen et al. [46]. According to Jiménez-Lobato et al. [26], *Datura* species have a very high chance of developing inbreeding depression in their native areas compared to areas where they have been introduced. A difference in herkogamy characteristics refers to the distance between the anther and the pistil. Reduced herkogamy (the distance between anthers and stigma) with flower position increases selfing, especially in low-nutrient and low-pollinator environments [47]. In *Datura*, selfing leads to inbreeding depression, which decreases plant resistance to herbivore attack [48].

A relatively high mutation rate can be produced by gamma ray radiation, inducing diversity in *Datura* species [49]. The genetic transformation of *Datura* was accomplished using the *Agrobacterium tumefaciens* C58 C1 Rig carrying the Ti plasmid pGV 2260 [50]. The ploidy profile of *Datura* plants grown in vitro will affect their genetic makeup [51]. In *Datura*, Indole Acetic Acid (IAA) (1 mg/L) is the best auxin for inducing somatic embryogenesis with a percentage of 80%, while Naphthalene Acetic Acid (NAA), 2,4-D, and Indole-3-Butyric Acid (IBA) do not induce somatic embryogenesis [52]. Exogenous Gibberellic acid (GA3) at concentrations between 10 ng/L and 1 mg/L accelerates the growth of *D. innoxia* hairy roots, elongation, and lateral branching [53]. Hyoscyamine concentrations were reduced by 35% under GA3 treatment, while 6-hydroxyhyoscyamine levels were boosted by 527% and scopolamine levels were increased by 400% [53]. In *Datura* spp., several transforming systems have been tested, including the *Agrobacterium*-mediated transformation system. A DNA-regenerated transgenic plant was transformed using protoplasts, leaf disks, and pollen, and the progeny displayed Mendelian inheritance. In addition, haploid transgenic plants have been regenerated. According to these findings, some components of the culture medium in which *Datura*-transformed root cultures are grown can significantly affect the proliferation and production of tropane alkaloids. It may be appropriate to use a two-step methodology, first in the presence of sucrose for increasing growth, as hyoscyamine and scopolamine can both be significantly increased when the level of this carbohydrate is altered, and then by removing some of the minerals from the medium, for example, by using specific compounds or by switching to an induction medium that is free from phosphorus and calcium.

In order to identify the interspecies genetic variations among these closely related varieties of *D. innoxia*, *D. metel*, and *D. arborea*, molecular markers of ITS4 and 5, matK, and rbcL genes are amplified and sequenced. In the study, *D. stramonium* isolate NN003 demonstrated an extremely close relationship, with a chloroplast genome similarity of 98%, 99%, and 99% [54]. Compared to the *D. arborea* variety, *D. metel* and *D. innoxia* were genetically closest. Barcode sequences of rbcL provided a higher level of resolution for the identification of clusters belonging to the same species. A study conducted by Prasad et al. [54] examined the conserved regions of the rcbL gene in three varieties of *D. innoxia*, *D. metel*, and *D. arborea* using BLAST+ (version 2.10.0). The relationships between *D. arborea* and *D. stramonium* were 98% and 94%, respectively, while the relationships between *D. innoxia* and *D. stramonium* were 95% and 95%, respectively. A close relationship exists between *D. metel* and *D. stramonium* (99%) and *D. innoxia* (97%), among others [54]. The taxonomic rearrangement of Egyptian *Datura* and the identification of novel taxa in the current study revealed their phylogenetic relationships. The morphological, molecular, and chemical results showed that the six Egyptian *Datura* genotypes exhibited 45%-79% similarity. Although *D. stramonium* is closely related to *D. tatula*, *D. inermis*, and *D. stramonium* (79, 57, and 60% similar), they exhibit significant differences up to (21, 43, and 40% dissimilarity, respectively) [55]. A chloroplast (cp) genome of *D. stramonium* is 155,871 bp in length, with 86,302 bp for the large (LSC) and 18,367 bp for the small (SSC), separated by a pair of inverted repeats (IRs), 25,601 bp) [56]. Due to different mutational pressures, the cp genome of *D. stramonium* may exhibit different genomic organization. The five most divergent coding regions and the four non-coding regions (trnH-psbA, rps4- trnS, ndhD-ccsA, and ndhI-ndhG) identified by whole plastome alignment can be used for phylogenetic and barcoding studies within the Solanaceae to develop molecular markers [56].

**Table 1:** Several studies have investigated the factors contributing to genetic variability in *Datura* species.

No.	Spesies	Study Sites	Genetic Variability Inducers	Results	Reference
1.	*D. innoxia*	India	*Agrobacterium*-mediated plant transformation	- The transformation of a zygotic embryo does not require wounding as it will result in the embryo’s death. - Plants without wounding exhibited a 54% transformation frequency. - Both transformation and regeneration of epidermal cells can be accomplished using epidermal cells.	[50]
2.	*D. innoxia*	France	In vitro micropropagation	- Explants of different types have different growth responses. - Several abnormal plants were produced when thin cell layers and internodes were planted on cytokinin-containing media. - Their morphology was different from that of the mother plant.	[40]
3.	*D. innoxia*	Punjab, Pakistan	Wastewater irrigation	- Plants grown with wastewater irrigation have a reduced biomass, resulting in a shorter average plant height.	[57]
4.	*D. innoxia*	Khyber Pakhtunkhwa (KP), Pakistan	Elevation gradient, Soil structure	- The lower elevation allowed *Datura* to produce a greater amount of biomass. - An individual’s morphological characteristics are affected by environmental factors, such as soil characteristics and bioclimatic layers.	[58]
5.	*D. innoxia*	Mexico	Pollination system among population from various location	- Pollination systems are affected by location differences. - Plant herkogamy (the position of the stigma) determines the level of outcrossing and selfing.	[38]
6.	*D. stramonium*	Mexico	Bioinformatics	- The genes associated with tropane alkaloid synthesis in *Datura* are *TRI*, *TRII*, *H6H*, and *PMT-10*.	[17]
7.	*D. stramonium*	California, USA	Agrobacterium-mediated plant transformation	- An increased rate of mutations is observed in transgenic plants grown through tissue culture, as indicated by mRNA sequencing and genome sequencing results. - No significant effect on gene expression.	[20]
8.	*D. stramonium*	Mexico and Spain	Flowers biology from different populations	- *Datura* populations introduced in Spain exhibit larger flower sizes than their Mexican counterparts.	[26]
9.	*D. stramonium*	Himachal Pradesh	Altitude differences	- GC-MS analysis reveals that *Datura* seeds contain alkaloid compounds, with important compounds such as scopolamine and atropine. - *Datura* plants at high altitudes produce higher amounts of scopolamine compared to those in lowland areas.	[39]
10.	*D. stramonium*	Indiana, USA	Natural selection	- Natural selection can cause a reduction in scopolamine levels. - This natural selection can be mediated by herbivores attacking *Datura* plants.	[59]
11.	*D. stramonium*	South Africa	Population study	- The outcrossing rate does not affect the number of plant populations.	[46]
12.	*D. stramonium*	Mexico	Different environment	- Flower characteristics are affected by differences in plant growth environments.	[47]
13.	*D. stramonium*	Teotihuaca’n, State of Mexico	Inbreeding	- Inbreeding reduces plant resistance to natural enemies, increasing damage by 4% from herbivore attacks and 8% from virus infections.	[48]
14.	*D. stramonium*	El Harrach, Algiers, Algeria	Gamma-irradiation on seeds	- Cobalt-60 to 60 Gy radiation dose did not give effect to decrease root and stem growth of plants. - 80 Gy dose caused slowing of root and stem growth of plants. - Germination rate was high, none of them were in the range of 30–50% (LD_50_).	[49]
15.	*D. stramonium*	Bulgarian	Differences in explants for in vitro culture	- The ploidy profile of plants grown in vitro will produce variations and differ from the mother plant.	[51]
16.	*D. stramonium*	Mexico	Cross breeding	- Herbivores are not necessarily resistant to plants containing high alkaloid concentrations.	[60]
17.	*D. stramonium*	Mexico	Adaptability to different environments	- Due to differences in herbivores around the population, natural selection has been driven by phenotypic differences in defensive traits in plants.	[61]
18.	*D. stramonium*	Mexico	Adaptability to different environments	- Variations are common in nature as a result of adaptation. - Variations in plant defense can be attributed to different pressures.	[62]
19.	*D. stramonium*	Italy, Portugal, Spain	Population and Temperature	- The best germination occurs when seeds are incubated at 16–24 °C. - Seeds incubated below 14 °C will have a lower germination rate.	[63]
20.	*D. stramonium*	Durham, North Carolina	Inbreeding depression	- Plants that have been crossed yield more viable pollen than plants that have selfed.	[64]
21.	*D. stramonium*	California, USA	Stigma-anther position	- The stigma position does not affect outcrossing in plants with stigma positions lower than the anthers, while the stigma position does affect outcrossing in plants with stigma positions higher than the anthers.	[65]
22.	*D. wrightii*	USA	Environment	- *D. wrightii*’s flower characteristics are influenced by its environmental conditions. - Various factors influence herkogamy variation, including trichome type, irrigation, and pests. - Watered plants have longer flower crowns. - Plants infested with pests produce fewer blooms.	[66]
23.	*D. wrightii*	Southern California, USA	Environment (irrigation)	- Plants that were unirrigated produced more glucose esters than those that were irrigated. - Irrigation increased leaf size without affecting trichome density. - The density of trichomes did not appear to be related to the production of acyl glucose ester.	[67]
24.	*D. wrightii*	California, USA	Herbivore and Methyl Jasmonate-Induced	- When plants are attacked by insects or treated with methyl jasmonate, they produce a higher level of volatile compounds.	[68]
25.	*D. metel*	Nagoya, Japan	Varieties	- *Datura* seeds and flowers contain more scopolamine and hyoscine than leaves.	[37]
26.	*Datura* spp.	Mexico	Population distribution	- *D. metel* is genetically related to *D. innoxia.*	[16]
27.	*Datura* spp.	Mexico	Taxonomy	- In Mexico, genus *Datura* were divided into 2 groups: (1) *D. innoxia*, *D. metel*, *D. lanosa*, and *D. wrightii*; and (2) *D. discolor*, *D. reburra*, *D. kymatocarpa* and *D. pruinosa*.	[34]

## 5. The Bioactive Compounds in the Genus Datura and Their Pharmacological Actions

*Datura metel* plants from North-Eastern India were investigated for their potential acaricidal effects by Kundu et al. [69]. *D. metel* extracts were analysed using UPLC-QTOF-ESI-MS to attempt the identification of tropane alkaloids, including tigloidin, hyoscyamine N-oxide, scopolamine N-oxide, tropinone, scopine, hyoscine, atropine, valtropine, ditigloyloxytropane, apohyoscine, tigloyloxytropane, norhyoscine, meteloidine, cuscohygrine, and tropine. As well as several flavonoids were identified, including luteolin-7-glucoside, catechin-3-O-rhamnoside, ferulic, quinic, syringic, sinapic acids, kempferol-3,7-O-diglucoside, naringenin-6-β-D-glucopyranoside, quercetin 3-O-glucosyl-xyloside, apigenin-7-O-glucoside, flavonol-3-glucoside, luteolin, epicatechin, chrysoeriol, diadzein, genistein, hesperidin, and hydroxycoumarin were also identified [69]. Kundu et al. [69] reported the maximum acaricidal activity to be exhibited by roots after 48 h of exposure with LC_50_ and LC_90_ values of 112.5 and 317.3 g/mL, respectively. A second phytochemical isolated from leaves of *D. stramonium* was tested using liquid chromatography quadrupole time of flight mass spectrometry (LC-QTOF-MS/MS) in Johannesburg (South Africa) [70]. Tapfuma et al. [70] found 76 compounds, such as eleganin, leucomycin A9, nocamycin I, nocardicin C, puromycin, neurymenolide B, jussiaeiine D, globomycin, kurarinone, mycinamicin II, obacunone, sreptonigrin, 3′-deoxyneamine, isocaorypalmine, linamarin, rhizobitoxine, prothiocarb, cinchoninone, imazaqulin, isosafrolepaeonianin E, entacapone, sarmentosin, eupatoroxin, etc.

**Figure 2:**
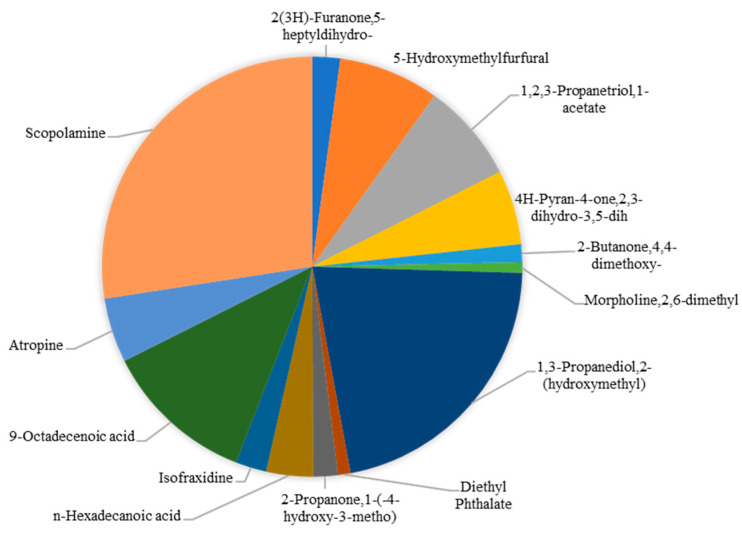
Compound profile of *Datura stramonium* seed extract using GC-MS [42].

According to Thakur et al. [39], *Datura* seed extracts using methanol contained at least 15 compounds detected using gas chromatography-mass spectrometry (GC-MS). An analysis of Thakur’s bioactive compounds is shown in Figure 2 as a pie chart. Scopolamine is the most abundant substance, with an area percentage of 27.06%. This is followed by 1,3-propanediol,2-(hydroxymethyl) (21.31%), 9-octadecenoic acid (11.53%), 5-hydroxymethylfurfural (7.33%), and 1,2,3-propanetriol,1-acetate (7.56%). *D. stramonium* leaves contain 1.55 mg/g dry weight of hyoscyamine and 1.55 mg/g dry weight of scopolamine [60]. It is well known that the *D. metel* plant contains a multitude of bioactive compounds, including saponins, alkaloids, steroids, tannins, flavonoids, and triterpenoids [71].

*Datura*’s defense against several attacking herbivores is aided by alkaloid compounds such as atropine and scopolamine [72]. *Datura*’s contains poisonous and hallucinogenic alkaloids, including atropine, hyoscine, and scopolamine [73]. In a recent study, it was found that several types of pests interfere with *Datura* species, including *Lema daturaphila*, *Epitrix parvula*, and *Sphenarium purpurascens* [62,67]. In *Datura* species, glandular trichomes protect from pest attacks [68]. It has been demonstrated that plants that receive sufficient air produce more trichomes than those that receive insufficient air [68]. In order to obtain a large number of trichomes, it is advised that *Datura* plants be irrigated with clean water so that they can produce larger amounts of bioactive compounds.

Scopolamine compounds are not only pest inhibitors but can also trigger flea beetle attacks due to their ability to stimulate flea beetle phagostimulation [59]. Additionally, De-la-Cruz et al. [60] point out that plants with a high level of alkaloids are not necessarily those with high density, but it depends on the specific alkaloids that are capable of controlling specific herbivores. *Datura* seeds and flowers contain more compounds, such as scopolamine, than their leaves [37]. The leaves of *Datura* plants produce alkaloid compounds and acyl glucose esters [68]. Several genes regulate the alkaloid compounds in *Datura*. A gene encoding the *hydroxylase of hydroxycyamine 6 β-nucleoside* (*H6H*) is involved in the synthesis of tropane alkaloids such as atropine and scopolamine, while the terpene synthase gene encodes *TPS-10* [17]. A particular group of plants known as tropane alkaloids is one of the oldest forms of plant medicine used by humans, with abundant amounts of these compounds being found in the Solanaceae family, the Erythroxylaceae, the Convolvulaceae, the Brassicaceae, and the Euphorbiaceae families [74,75].

Anisodamine, atropine (hyoscyamine), and scopolamine are the main tropane alkaloids present in *D. stramonium* [76,77]. In the past, scopolamine and atropine have been used for the treatment of asthma, rheumatism, and spasticity [78]. It has been identified that scopolamine is one of the essential active medicinal compounds according to the World Health Organization (WHO) [79]. As an alkaloid, scopolamine is the primary bioactive compound. Several conditions necessitate caution when using scopolamine or frequent monitoring of its effects. On the other side, scopolamine worsens psychosis. Acute toxic psychosis, agitation, speech disorder, hallucinations, paranoia, and delusions have also been reported [80]. Blurred vision, dilated pupils, and dry mouth may result from the use of Scopolamine patches. The majority of vision disturbances are caused by inadequate handwashing techniques after patch application [81]. *D. metel* is also rich in withanolides, and its flowers provide pain relief [82] and also have hallucinogenic properties. Besides being used as a substitute for opium, the seeds of this plant are also known to relieve dental pain due to the fact that chewing its leaves can treat cavities [83,84]. *Datura* has wide applications in Ayurvedic medicines. *Datura* contains many constituents that contribute to the treatment of hair fall and other skin disorders [85]. In some cases, bleeding disorders may be treated with the seeds of *D. metel*. It has also been shown that the plant possesses antimicrobial and antiinflammatory properties [7]. In addition to dilation of the pupil, atropine (one of the key components of *D. metel*) is useful in eye surgery [86]. Several studies have indicated that *D. metel* plant extracts have herbicidal properties, which can remove unwanted weeds by extracting methanol from the dried leaves [87].

In an acetic acid test with Albino mice, *D. fastuosa* L. leaf extract showed an analgesic effect when combined with acetic acid (0.6% (10 mL/kg^−1^)) on days 7, 14, and 21 after an 800 mg/kg injection of the extract [88]. The rectal temperature and apomorphine hypothermia were significantly reduced with an increase in water intake of leaf extract [88,89]. An aqueous extract of *D. fastuosa* produced neuropsychopharmacological effects in Wistar rats and Albino mice. As a result of treatment with seeds and leaves extracts at doses of 400 and 800 mg/kg, researchers observed an increase in motor activity, a reduction in barbiturate sleeping duration, ptosis induced by haloperidol, antagonized catalepsy, and immobility due to forced swimming [88]. The results of clinical trials have demonstrated that *Datura* extract has antidepressant properties at low doses. The alkaloid scopolamine (a component of the plant that stimulates the central nervous system) may have antidepressant effects on the central nervous system [89].

The phytochemical and antioxidant potentials of methanol and ethanol extracts of stems, leaves, and roots of *Datura* are briefly summarized in Table 2. A number of antinociceptive targets have been identified as promising for the treatment of inflammatory pain, and these include cannabinoid receptors 1, predominantly, as well as non-cannabinoid receptors, such as PPAR alpha and 5-HT 1B [90]. In addition to its effects on nitric oxide, daturaolone may also act on other mediators of depression. In the monoamine theory of depression, depression is a manifestation of disturbed neurotransmission caused by stress, specifically the monoamines responsible for transmission of signals, i.e., norepinephrine, dopamine, and serotonin [91]. Moreover, daturaolone possesses a variety of molecular targets, which is in agreement with previous studies that have demonstrated the interrelated effects of terpenoids on angiogenesis, inflammation, nociception, and depression. Compounds have different efficacies depending on their activities.

**Figure 3:**
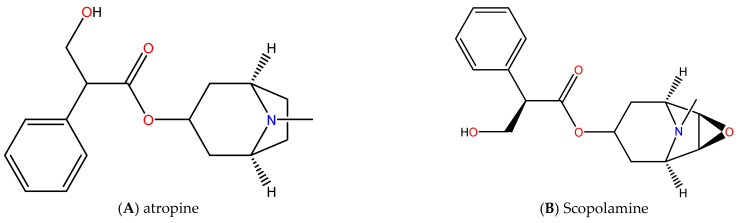

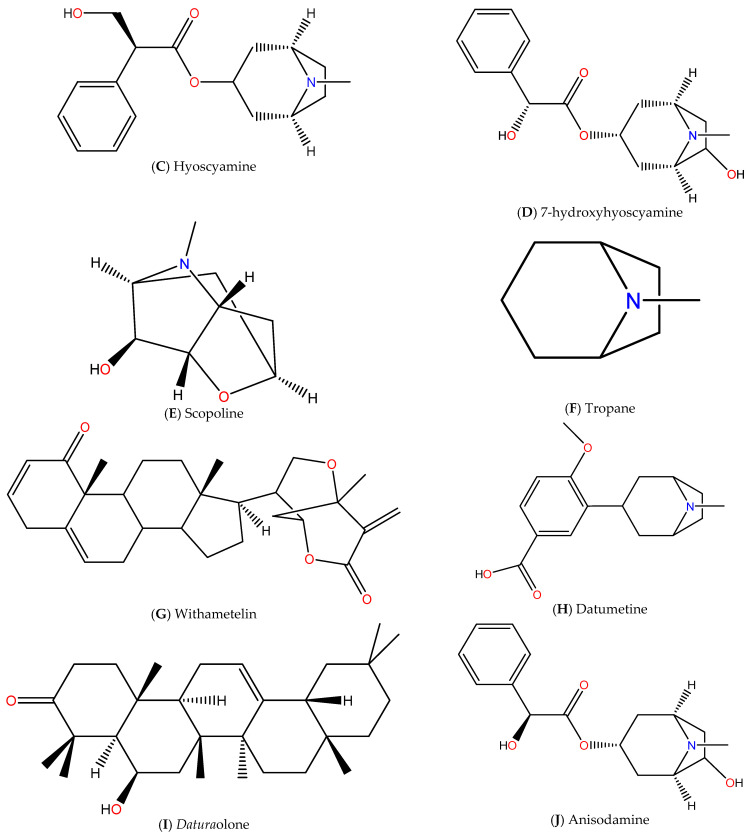
Structures of the major bioactive compounds in genus *Datura* [93,94,95,96,97,98,99,100,101,102].

According to the above information about *Datura* species and their parts, numerous significant bioactive compounds have been identified throughout *Datura* species. In Figure 3, we can briefly view the chemical structure of *Datura*’s major compounds, for instance, atropine, scopolamine, hyoscyamine, 7-hydroxyhyoscyamine, tropane, withametelin, datumetine, daturaolone, and anisodamine. Besides being used for several ailments, the *Datura* plant is extremely toxic. Despite its toxic effects, it can be effective in treating a wide range of medical conditions. A higher dose of *Datura* extract, which contains atropine and scopolamine, can cause central nervous system damage, hallucinations, convulsions, arrhythmias, irregular heartbeats, dementia, blurred vision, dry mouth, coma, and even death [92]. Considering this toxicity, extracting and purifying should be done carefully. It is also challenging to isolate and purify a specific compound that interacts inside the plant and develop reliable animal models for *Datura*-related toxicity. Thus, extensive research and clinical trials are required to separate toxic and non-toxic chemical compounds to and evaluate their therapeutic safety. A further investigation is needed to understand their biosynthesis pathways and the genetic regulation of these bioactive compounds. The development of novel drugs and treatments derived from *Datura* could be made possible by understanding these mechanisms.

**Table 2:** Compilation of existing studies on other bioactive compounds found in the genus *Datura* and their pharmacological effects.

No.	Datura Species	Datura Source	Pharmacological Activity	Bioactive Compound	Result	Reference
1.	*Datura innoxia* Mill.	Fruits, steams, leaves	Brine shrimp lethality assay, anticancer in human leukimia (THP-1) cell line, protein kinase inhibitory assay, antifungal, antibacterial	- Methanol chloroform leaf extract (catechin and apigenin). - Methanol leaf extract (catechin, apigenin, quercetin). - Distilled water stem extract (rutin, caffeic acid, catechin, myrecetin, quercetin). - n-hexane fruit extract (flavonoid) - Chloroform fruit extract (catechin, apigenin). - Ethanol fruit extract (catechin, apigenin, myrecetin, quercetin, kaempferol).	- Cytotoxicity against brine shrimps classified 25% of the leaves, 16% of the stem, and 8.3% of the fruit extracts as highly toxic (LD_50_ ≤ 100 μg/mL). - An IC_50_ of 4.52 and 3.49 g/mL for chloroform and n-hexane fruit extracts against human leukemia (THP-1) cells was observed, respectively. - *Aspergillus niger* exhibits significant antimicrobial activity against leaf extracts of *Micrococcus luteus* and fruit extracts of n-hexane (MIC 3.70 and 12.5 μg/mL, respectively).	[103]
2.	*Datura innoxia*	Leaves	Antioxidant, enzyme inhibitor	- The main flavonoids identified were (+) catechin and (−) epicatechin and hyperoside. (+) Catechin and (−) epicatechin showed very high concentrations in *D. innoxia* (12937.39 ± 108.86 and 24147.64 ± 2512.35 μg/g of dry plant, respectively). - Total phenolic amount was higher in *D. innoxia* (73.80 ± 0.57 mg GAE/g extract). - Caffeic acid 17.58 ± 1.24 μg/g extract, quercetin 9.47 ± 0.14 μg/g extract, gallic acid 3.66 ± 0.41 μg/g extract, protocatechuic acid 62.15 ± 0.72 μg/g extract.	- The highest radical scavenging activities using DPPH and ABTS radicals with value 534.55 ± 4.88 mg TE/g extracts. - The strongest activity against α-amylase (356.35 ± 4.60 mg ACEs/g extracts) and tyrosinase 317.03 ± 2.00 mg KAEs/g extracts). - It has values of 329.21 ± 2.27 mg ACEs/g extracts against α-amylase and 267.48 ± 6.58 mg KAEs/g extracts (against tyrosinase).	[104]
3.	*Datura stramonium*	Leaves	Anticancer in MCF-7, MDA-MB 231 (breast cancer cell lines), PC-3 (prostate cancer cell lines), brine shrimp lethality assay, protein kinase inhibitory assay	- Rutin 0.89 ± 0.03 μg/mg, gallic acid, 0.35 ± 0.07 μg/mg, catechin 0.24 ± 0.02 μg/mg, apigenin 0.29 ± 0.09 μg/mg.	- DSL-EA (*Datura stramonium* ethyl acetate) showed mediocre cytotoxic potential with 14.34% inhibition against PC-3, 37.67% in case of MDA-MB 231 and 40.56% against MCF-7 cell line. - Brine shrimp lethality assay in DSL-EA showed significant results with LC_50_ values of 12.04 ± 0.85 μg/mL. - DSL-EA exhibited a bald zone of 12.50 mm at MIC: 100 μg/disc.	[105]
4.	*Datura innoxia*	Leaves	Anticancer in MCF-7, MDA-MB 231 (breast cancer cell lines), PC-3 (prostate cancer cell lines), brine shrimp lethality assay, protein kinase inhibitory assay	- Rutin 0.036 ± 0.004 μg/mg, caffeic acid 0.27 ± 0.03 μg/mg.	- DIL-EA (*Datura innoxia* ethyl acetate) against each cancer cell line with IC_50_ values of 2.86 ± 0.1 μg/mL against PC-3, 1.56 ± 0.16 μg/mL against MDA-MB 231 and 2.45 ± 0.04 μg/mL in case of MCF-7 cell line. - Brine shrimp lethality assay in DIL-EA showed significant results with LC_50_ values of 10.37 ± 1.56 μg/mL - DIL-EA showed significant inhibitory potential by exhibiting a 19 mm bald phenotype zone at a concentration of 100 μg/disc (MIC: 25 μg/disc).	[106]
5.	*Datura innoxia*	Leaves	Anticancer, antioxidant, antiinflammatory	Withametelin	- Cytotoxic to cancer cells (DU145 IC_50_ 7.67 ± 0.54 mM) than normal lymphocytes (IC_50_ 33.55 ± 1.31 mM). - Rreduced inflammatory paw edema (68.94 ± 5.55%), heat-induced pain (78.94 ± 6.87%) and immobility time (50%) in animals.	[107]
6.	*Datura metel*	Leaves	Antiinflammatory	Sesquiterpenoids	- The compound 5 possessed the best inhibitory effect as anti-inflammatory activity against the production of nitrogen oxide in lipopolysaccharide-induced RAW264.7 cells, with the IC_50_ value reaching 9.33–11.67 μM, which was lower than positive control, L-NMMA, with IC_50_ range from 13.64 to 17.02 μM.	[12]
7.	*Datura innoxia*	Leaves	Antiinflammatory	Daturaolone	- Daturaolone significantly inhibited NF- B and nitric oxide production with IC_50_ values of 1.2 ± 0.8 and 4.51 ± 0.92 μg/mL, respectively. - Daturaolone significantly reduced inflammatory paw edema (81.73 ± 3.16%), heat-induced pain (89.47 ± 9.01% antinociception) and stress-induced depression (68 ± 9.22 s immobility time in tail suspension test).	[108]
8.	*Datura innoxia*	Leaves	Acute and subacute oral toxicity in Sprague Dawley rats	Withametelin and daturaolone	- Subacute daily dose of withametelin was 5, 2.5, and 1.25 mg/kg but, for daturaolone, it was 10, 5, and 2.5 mg/kg. - High dose (5 and 2.5 mg/kg) withametelin groups showed dose dependent changes in the general, hematological, biochemical and histopathological parameters. - In both sexes, the most prominent being hyperthyroidism while no toxicity was observed at lower doses (1.25 and 0.75 mg/kg), No Observable Adverse Effect Level (NOAEL) being 1.25 mg/kg. - Daturaolone safer and showed dose dependent significant changes in hepatic enzyme (Alanine Transaminase), bilirubin, creatinine, and glucose levels while histological changes in testes were also observed. - Lower doses (5, 2.5, and 1.25 mg/kg) of daturaolone showed no significant toxic effects and 5 mg/kg was declared as its NOAEL.	[109]
9.	*Datura fastuosa*	Roots	Antioxidant	- 1-methylpyrrolidine-2-carboxylic acid, 8-azabicyclo[3.2.1]octan-3-ol,8-methyl-,endo-,4-bromo-7-chloro-8-fluoro-2-phenylquinoline, atropine, [2,3-diacetyloxy-5-(2,4,6-triacetyloxy- 3-chlorophenoxy)phenyl] acetate, octadecanoic acid, beta-amyrin, (2R,3S)-2-(3,4dihydroxyphenyl)-3,4-dihydro-2H-chromene 3,5,7-triol, 3-dimethylamino-2-methyl-2-propinal, 12-octadecadienoic acid (Z,Z)-1-hexadecyne, scopolamine, propenoic acid-2-phenyl-,8-methyl 8-azabicyclo[3.2.1]octan-3-ylester, 8 azabicyclo[3.2.1]octane-3,6-diol, diacetate (ester), [1R-[1.alpha.,3.beta.(E),5.alpha.6.alpha.]]-, 2 butenoic acid, 2-methyl-,8-methyl-1-6-(1 oxopropoxy)-8-azabicyclo[3.2.1]oct-3-yl ester. - The root extract contains a variety of major compounds, including phenolics, flavonoids, alkaloids, steroids, fatty acids, esters, hydrocarbons, terpenoids, aldehydes, and ketones.	- The methanol crude extract demonstrated antioxidant potential compared to standard ascorbic acid, exhibiting DPPH scavenging activity.	[110]
10.	*Datura stramonium*	Leaves	Anticandidal	- GC-MS analysis of the F8 fraction indicated the presence of 23 compounds, with the major compounds being Phthalic acid, di (2-propylpentyl) ester (Compound 1), Pentadecane (Compound 2), Octadecane (Compound 3), Benzoic acid, 3-Amino-5-Hydroxy-, Methyl ester (Compound 4), and 1,2-Benzenedicarboxylic acid, bis (2-ethylhexyl) ester (Compound 5).	- The highest zones of inhibition against *Candida guilliermondii* (20.33 ± 0.56 mm). - *Candida tropicalis* (16.33 ± 0.58 mm), and *Candida albicans* (14.66 ± 1.05 mm), with a minimum inhibitory concentration (MIC) value of 25 μg/mL.	[111]
11.	*Datura metel*	Leaves	Antibacterial and antioxidant	- The qualitative phytochemical analysis revealed the presence of steroids, alkaloids, phenolic compounds, cardiac glycosides, flavonoids, saponins, tannins, anthraquinones and coumarins in methanolic crude extracts.	- The strongest antibacterial activity against *Salmonella typhi*, *Acinetobacter baumannii*, *Klebsiella pneumoniae*, and methicillin-resistant *Staphylococcus aureus* (MRSA). - Antimicrobial activity against the tested organisms, with the average diameter of the zone of inhibition ranging 19.21 mm. - In antioxidant assays, with 78.3 ± 2% inhibition and an IC_50_ value of 40.1 ± 4 µg/mL.	[112]

## 6. The Role of Biosynthesis Genes in Metabolic Regulation Across Datura Species

The secondary metabolism of plants involves highly conserved biosynthetic pathways as well as complex genetic machinery to produce a range of chemical compounds that play essential roles in the eco-physiology of the plant. In biotic interactions, secondary metabolites may serve as adaptations to deal with natural enemies of plants, producing toxic or deterrent effects on their consumers [113,114,115]. Specialized metabolic pathways produce plant metabolic diversity. A variety of biosynthetic gene pathways are involved in the synthesis of specialized metabolites. Several metabolic pathways colocalize with biosynthesis genes, which promotes coregulation and localized synthesis of metabolites. A genome annotation of tropinone reductase I (TRI), the enzyme responsible for producing tropane alkaloid compounds in *Datura stramonium*, has been assembled and annotated. It was found that *D. stramonium* duplication significantly enriched the biosynthesis of polyamines, including putrescine, a precursor molecule for tropane alkaloids [116]. Thus, it can be concluded that the tropane alkaloid biosynthesis pathway is quite well characterized in the next generation based on the number of copies of the tropane alkaloid biosynthesis gene [20]. The tropinone reductase enzyme converts pseudotropin into tropinone reductase II (TRII) for pharmacologically important alkaloids atropine and scopolamine [117]. Secondary metabolites produced through cell culture or plant organs are the best alternative to whole plant material. This increases the production of secondary metabolites in in vitro systems. The provision of precursors or intermediate metabolites is a clear approach and has a high success rate. The effect of providing different precursors, such as tropisone, on *D. innoxia* Mill root cultures showed an increase in tropane alkaloids [117].

The *Datura* transcriptomes show several enriched protein families (significantly more from the full protein set), with different signals of expansion, physicochemical divergence, and/or positive selection. *D. stramonium* was the only organism with expanding and positive selection of UDP-dependent glycosyl transferase. Abiotic and biotic stresses (e.g., herbivore attack) prompt UDP-glycosylases to glycosylate various phytohormones and metabolites in plants [118,119]. All *Datura* species also contain enriched protein families related to glycosyltransferases and oxidoreductases. A transferase and an oxidoreductase are enzymes that are directly involved in terpene biosynthesis, tropane alkaloids [120,121], as well as many other plant defensive compounds via glycosylation, including phenolics, glucosinolates, salicylates, and anthocyanins [121]. A decrease in glycosyltransferase activity was observed in all the species except *D. stramonium*, whereas an increase in oxidoreductases was observed only in *D. pruinose* and *D. stramonium* [122]. Additionally, *D. pruinose* and *D. wrightii* both displayed positive selection of terpenoid proteins, but *D. innoxia* and *D. stramonium* did not [122].

Tropane alkaloids are synthesized by proteins from families expanded in the *Datura* branch, and proteins that contain positively selected conserved amino acids (codons). The enzymes Cytochrome P450 (IPR001128), Transferase (IPR003480), NADH: ubiquinone oxidoreductase (IPR003918), and Phosphoethanolamine N-methyltransferase (IPR025771) rearrange littorine (a tropane alkaloid) to produce atropine, hyosciamine, and scopolamine [123]. Scopolamine is biosynthesised via a gene called *H6H*, a member of the *Hyoscyamine (6S)-dioxygenase* family [123]. A number of these enzymes participate in tropane alkaloid biosynthesis, which can be observed in positively selected proteins with divergent physicochemical properties [124]. *Datura*’s last common ancestor was only one gene, whereas *D. stramonium* from Ticumán and Teotihuacán in Mexico contain three and two gene copies, respectively [125]. *PMT* is a key gene that catalyzes the formation of N-methylputrescine from putrescine and S-adenosyl-L-methionine, which leads to the production of hygrine and other tropane alkaloids [125]. It was discovered that the Pfam annotation of the *PMT* genes in the Ticumán genome revealed that the homolog of the spermine synthase in the Ticumán genome contains an extra domain compared to its homolog in the Teotihuacán genome [125]. Among a variety of environmental stresses, including herbivory and pathogenesis, Kasukabe et al. [126] found that overexpression of spermidine synthase enhanced tolerance.

## 7. Conclusions

This study has concluded that genetic variability in *Datura* is caused by natural factors, including natural selection as a result of environmental influences, predators and pests in the *Datura* growing area, plant crossing, and induction of diversity through genetic transformation and tissue culture. *Datura* species can be genetically altered, which can affect their concentration and efficacy of bioactive compounds. Additionally, these alterations can lead to new pharmacological activities, opening new therapeutic opportunities. Nevertheless, the variability of their composition may pose challenges to standardizing plant-based medicines. Understanding genetic mechanisms underpinning these alterations is needed to harness their potential benefits effectively in the future. Standardizing plant-based medications could also be improved by developing methods to stabilize compound concentrations. Identifying genes involved in secondary compound synthesis and genes related to defense, among other functions, is essential for elucidating their roles in species divergence. Physicochemical divergences in enzymes and proteins related to tropane alkaloids were revealed in *Datura* gene species. An analysis of gene families can help understand species’ genomic evolution and economic and ecological implications. Considering functional analysis, overrepresented candidate genes with positive selection signals and physicochemical divergence should be tested in further research. This will provide us with a better understanding of their significance in both ecological and evolutionary terms.

## Data Availability

This article provides detailed information about the data presented in this study.
